# Comparison of Corn and Rice Sensitivity to Cyanobacterial Extract and Anatoxin-a in Soil and Hydroponic Media

**DOI:** 10.3390/plants15182803

**Published:** 2026-09-13

**Authors:** Clare T. Muller, Scott A. Heckathorn, Jennifer K. Boldt

**Affiliations:** 1 Department of Environmental Sciences, University of Toledo, Toledo, OH 43606, USA; scott.heckathorn@utoledo.edu; 2 Agricultural Research Service, United States Department of Agriculture, Toledo, OH 43606, USA; jennifer.boldt@usda.gov

**Keywords:** anatoxin-a, cyanotoxins, harmful algal blooms, photosynthesis

## Abstract

Cyanobacterial toxins (cyanotoxins) produced in harmful algal blooms are toxic to eukaryotic cells. Plants exposed to cyanotoxins through irrigation can accumulate toxins and have decreased yields, so an understanding of the effects of cyanotoxins on major crop plants is important. Although the effects of some cyanotoxins on growth and photosynthesis of common agricultural crops have been studied, how cyanotoxin uptake may be impacted by differences in root anatomy has not been studied. Hence, we exposed corn and rice, plants lacking and possessing a constitutively well-developed root exodermis, respectively, to cyanobacterial extracts or pure anatoxin-a to test how root structure impacts cyanotoxin sensitivity. We conducted three comparative experiments: (1) a pot study in which corn and rice were watered with cyanobacterial lysed-cell extracts, (2) a hydroponic study wherein plants were treated with cyanobacterial extracts, and (3) a study wherein plants were grown in pots and watered with pure anatoxin-a. We hypothesized that the constitutive exodermis produced by rice would make rice plants less sensitive to cyanotoxins than corn. However, for all three experiments, photosynthesis, chlorophyll, and growth of corn and rice plants were reduced when roots were exposed to either extracts or anatoxin-a, compared to non-treated controls, although corn plants were affected sooner and/or more dramatically than rice. Our results indicate the root exodermis of rice did not prevent cyanotoxin uptake by roots nor prevent cyanotoxin effects.

## 1. Introduction

Cyanobacterial harmful algal blooms (cHABs) are frequent seasonal occurrences in freshwater ecosystems [1]. Due to the production of toxins (cyanotoxins) by cyanobacterial species that form blooms, e.g., *Microcystis aeruginosa* and *Anabaena flos-aquae*, cHABs are a concern for public health, recreational land use, public revenue, and aquatic ecology [2,3]. More recently, the possibility of exposing agricultural plants to cyanotoxins via irrigation with cHAB-contaminated waters is of increasing concern [4]. For example, anatoxin-a, a product of multiple bloom-forming cyanobacterial species including *A. flos-aquae*, is a potent agonist of acetylcholine receptors in animal nervous systems and can be present at high levels in cHAB waters [5]. Mitigating the occurrence and toxicity of cHABs is a complex problem for people managing freshwater supplies and for farmers [6], so understanding how agricultural plants respond to cyanotoxin exposure is important.

Cyanotoxins are known to inhibit plant photosynthesis and growth [7,8,9]. For photosynthesis, damage to photosystem II, lower rubisco activity, and loss of chlorophyll are the main effects of some cyanotoxins [10], which lead to lower net carbon fixation rates and overall rates of photosynthesis. Exposure to cyanotoxins over multiple weeks or at high concentrations can reduce plant growth [10,11,12,13]. Depending on the study, either roots [14,15] or shoots [16,17] were more severely affected by cyanotoxin exposure. Most studies to date that have examined the effects of cyanotoxins on plants have focused on growth, yield or bioaccumulation [18,19,20,21,22]; however, the mechanism for uptake and transport of most cyanotoxins and cyanobacterial metabolites in plants is unknown. Whether differences in root structure (e.g., presence or absence of a well-developed exodermis) among plants affect their sensitivity to cyanotoxins is yet to be determined.

Corn (*Zea mays*) and rice (*Oryza sativa*) are two of the world’s most important food crops [23,24]. They differ functionally in a number of ways: corn is a C_4_ plant suited to drier conditions than rice, is flooding intolerant, and produces aerial roots [25]. In contrast, rice roots constitutively possess a thick suberized exodermis which limits water uptake into the root during soil flooding—a feature normally lacking in corn [26,27]. It is possible that the exodermis in rice increases tolerance to cyanotoxins in the soil, and there is some evidence to suggest that corn is less tolerant of cyanotoxins compared to rice [8]; however, this has not been tested directly. To our knowledge, no studies have compared cyanotoxin sensitivity in species with and without a well-developed root exodermis.

The functional and anatomical differences in the roots of corn and rice, despite both being cereal grasses and field crops, make for an interesting direct comparison of their responses to cyanotoxin exposure. So, we grew corn and rice in soil and hydroponically with and without cyanobacterial cell extracts or the purified cyanotoxin, anatoxin-a, and compared their growth, photosynthetic, and root responses to cyanotoxins. Because of the lack of a constitutive exodermis, we hypothesized that corn would be more sensitive to cyanotoxin exposure at the root level than rice plants that constitutively possess a well-developed exodermis.

## 2. Results

### 2.1. Experiment 1: Cyanobacterial Extracts in Soil

**Figure 1:**
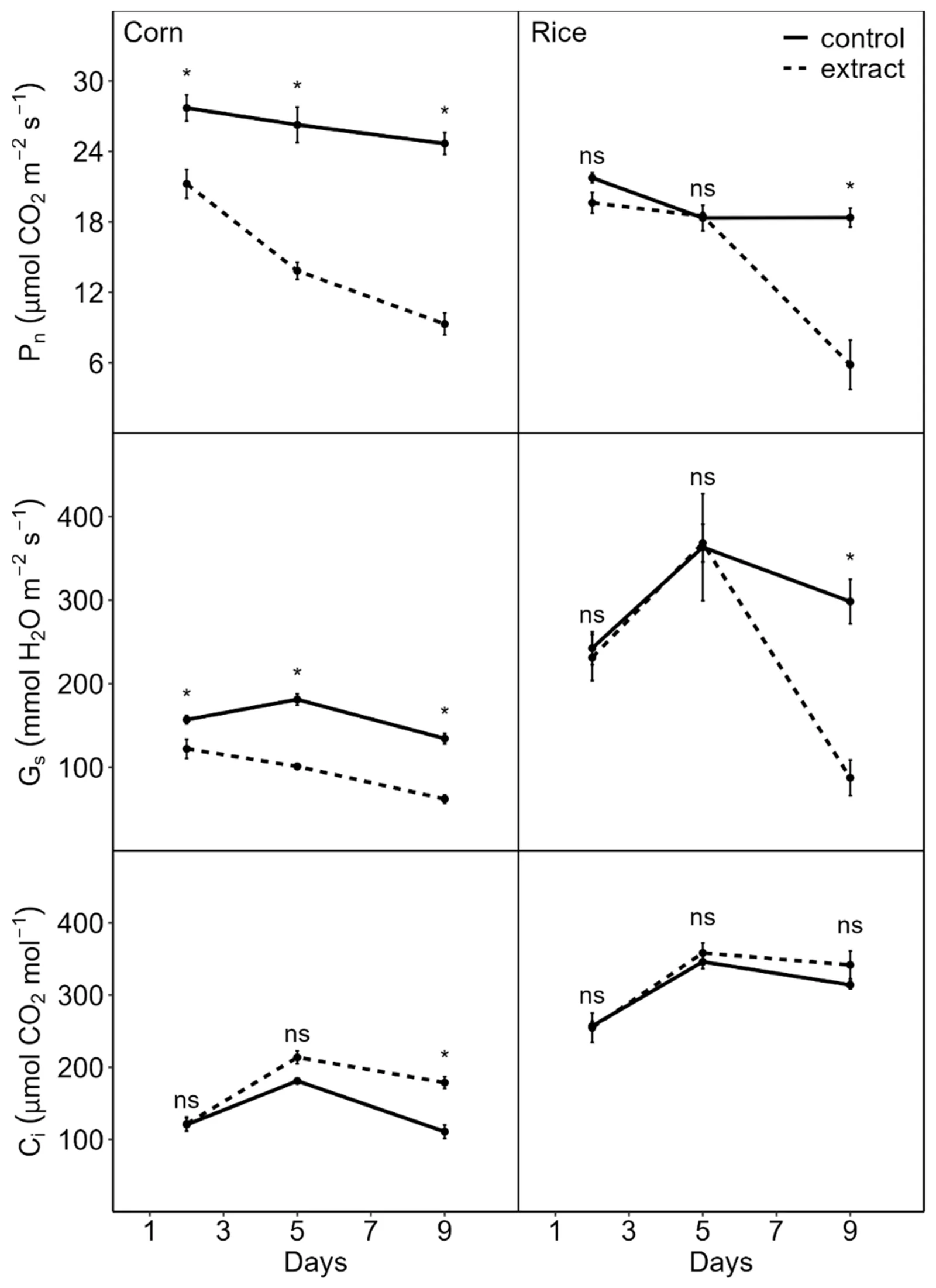
Gas-exchange measurements for corn and rice plants grown in soil and watered with nutrient solution (control) or lysed *Anabaena flos-aquae* cells in nutrient solution (extract) at three time points. Values are means ± 1 SE for net photosynthesis (P_n_), stomatal conductance (G_s_), and leaf internal CO_2_ concentration (C_i_). Asterisks represent significant differences (*p*-value ≤ 0.05) between control and extract treatment means for each time point based on Welch’s *t*-tests; ns = non-significant.

Corn and rice plants grown in soil watered with cyanobacterial extracts had decreased net photosynthesis (P_n_) and stomatal conductance (G_s_), compared to control plants, on all three measurement days (Figure 1). For corn, P_n_ was 23% lower for plants watered with extract after 2 d of treatment and 62% lower, compared to controls, by the end of the experiment (12 d); G_s_ was also lower within 2 d and declined further over subsequent days. For rice, P_n_ was unaffected by treatment after 2 d and 5 d, but it was 68% lower after 9 d for extracts compared to controls; similar effects on G_s_ were observed. Both species had similar internal CO_2_ concentrations (C_i_) between control plants and those watered with extracts for the duration of the experiment, except for corn plants after 9 d, in which C_i_ was 61% higher in extract plants compared to controls. In general, when P_n_ and G_s_ both decrease, but C_i_ does not, the decrease in G_s_ matches the decrease in P_n_. When P_n_ and G_s_ both decrease, but C_i_ increases, then the decrease in CO_2_ fixation exceeds stomatal limitations to P_n_. Hence, in this experiment, the effects of the cyanobacterial extracts on CO_2_ fixation played a greater role than G_s_ in decreasing P_n_ in both corn and rice.

**Figure 2:**
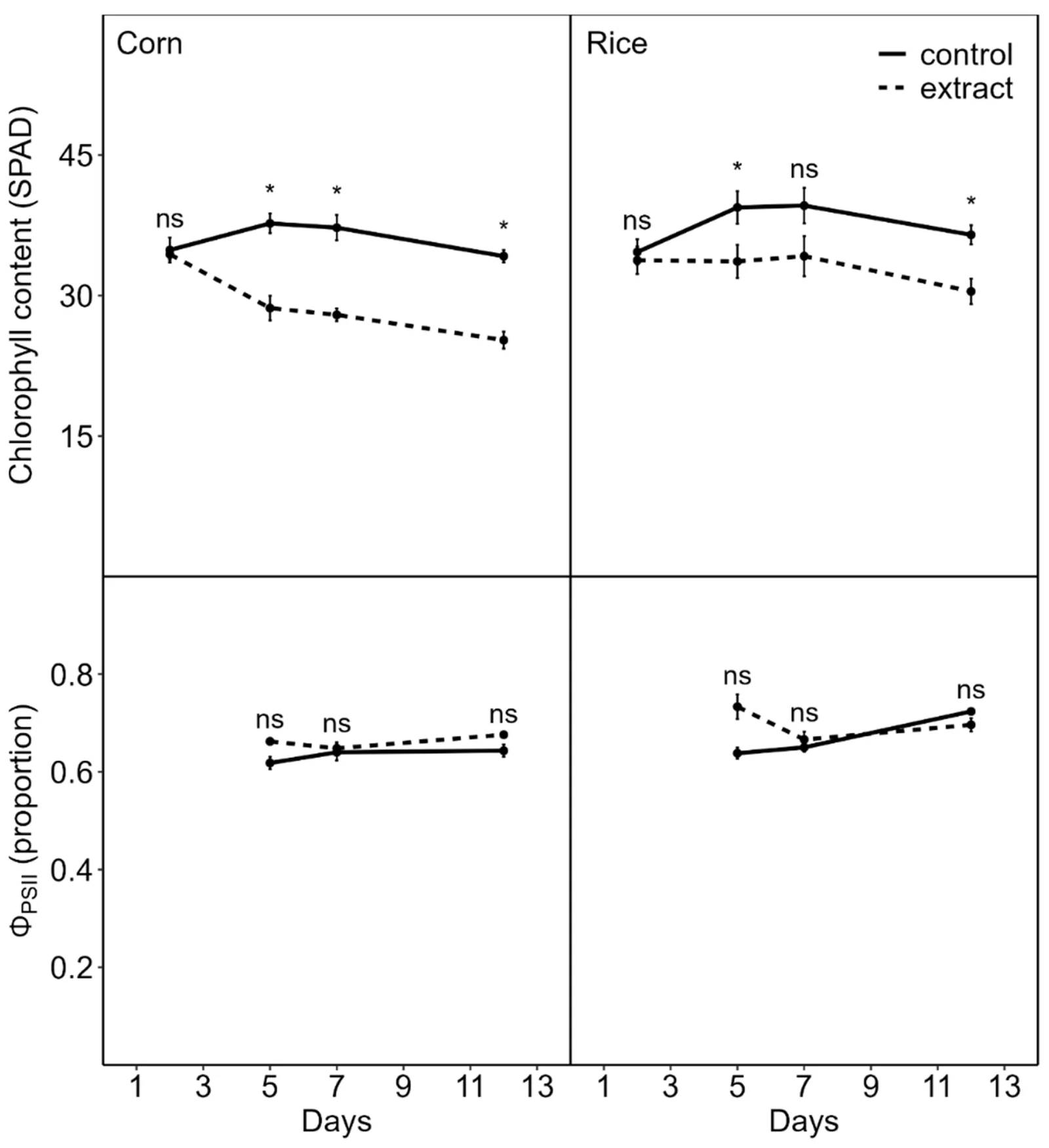
Chlorophyll concentration and photosystem-II yield (Φ_PSII_) of corn and rice plants grown in soil and watered with nutrient solution (control) or lysed *Anabaena flos-aquae* cells in nutrient solution (extract) at three time points. Values represent means ± 1 SE. Asterisks represent significant differences (*p*-value ≤ 0.05) between control and extract treatment means for each time point based on Welch’s *t*-tests; ns = non-significant.

Declines in chlorophyll content (relative concentration per unit area) were first detected in both species after 5 d of treatment for plants watered with extracts (Figure 2). By 12 d, corn plants watered with extract were 26% lower in chlorophyll content than controls, and rice plants watered with extract were 17% lower than controls. Photosystem-II yield (Φ_PSII_) was not significantly different between extract and control plants for either species for the duration of the experiment (Figure 2).

**Figure 3:**
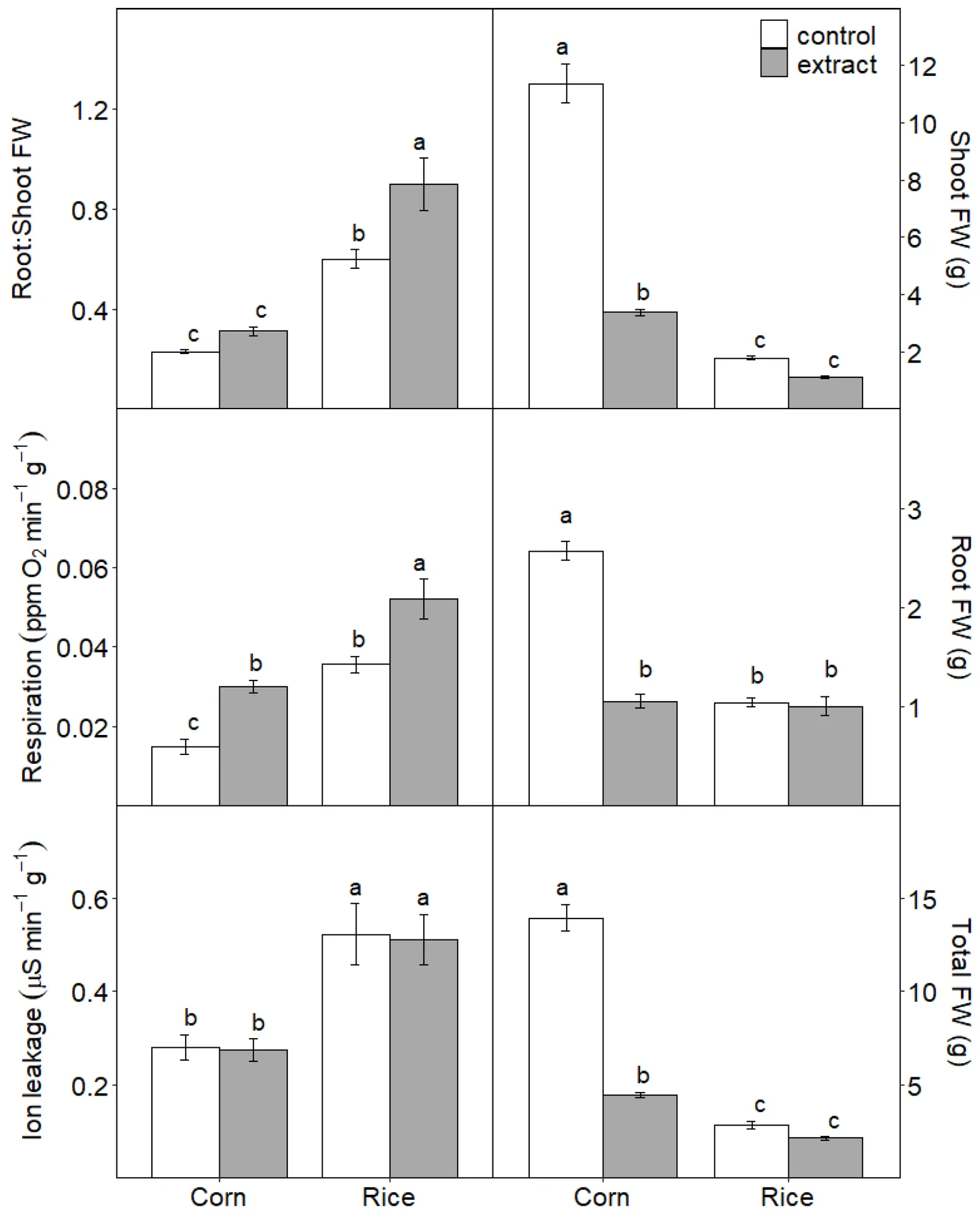
Biomass (as fresh weight, FW) and root functional measurements of corn and rice plants grown in soil and watered with nutrient solution (control) or lysed *Anabaena flos-aquae* cells in nutrient solution (extract) for 12 days. Bars represent means ± 1 SE. Different letters within each graph represent pairwise differences in means among species and treatment combinations for each response variable.

At the end of the experiment, shoot, root, and total fresh weight (FW) were lower in corn plants watered with extracts compared to controls; however, the biomass of rice plants was not significantly affected by treatment with extract (Figure 3). In contrast, the root-to-shoot mass ratio increased significantly in rice plants watered with extracts compared to controls, driven by a relatively larger (non-significant) decrease in shoot mass coupled with a smaller non-significant decrease in root mass; root-to-shoot mass ratio did not increase significantly in corn (Figure 3). Root respiration increased significantly in both species in plants watered with extracts compared to controls: 102% higher respiration in corn and 46% higher in rice (Figure 3). Ion leakage from roots was not significantly different between treatments in either species (Figure 3).

### 2.2. Experiment 2: Cyanobacterial Extracts in Hydroponics

**Figure 4:**
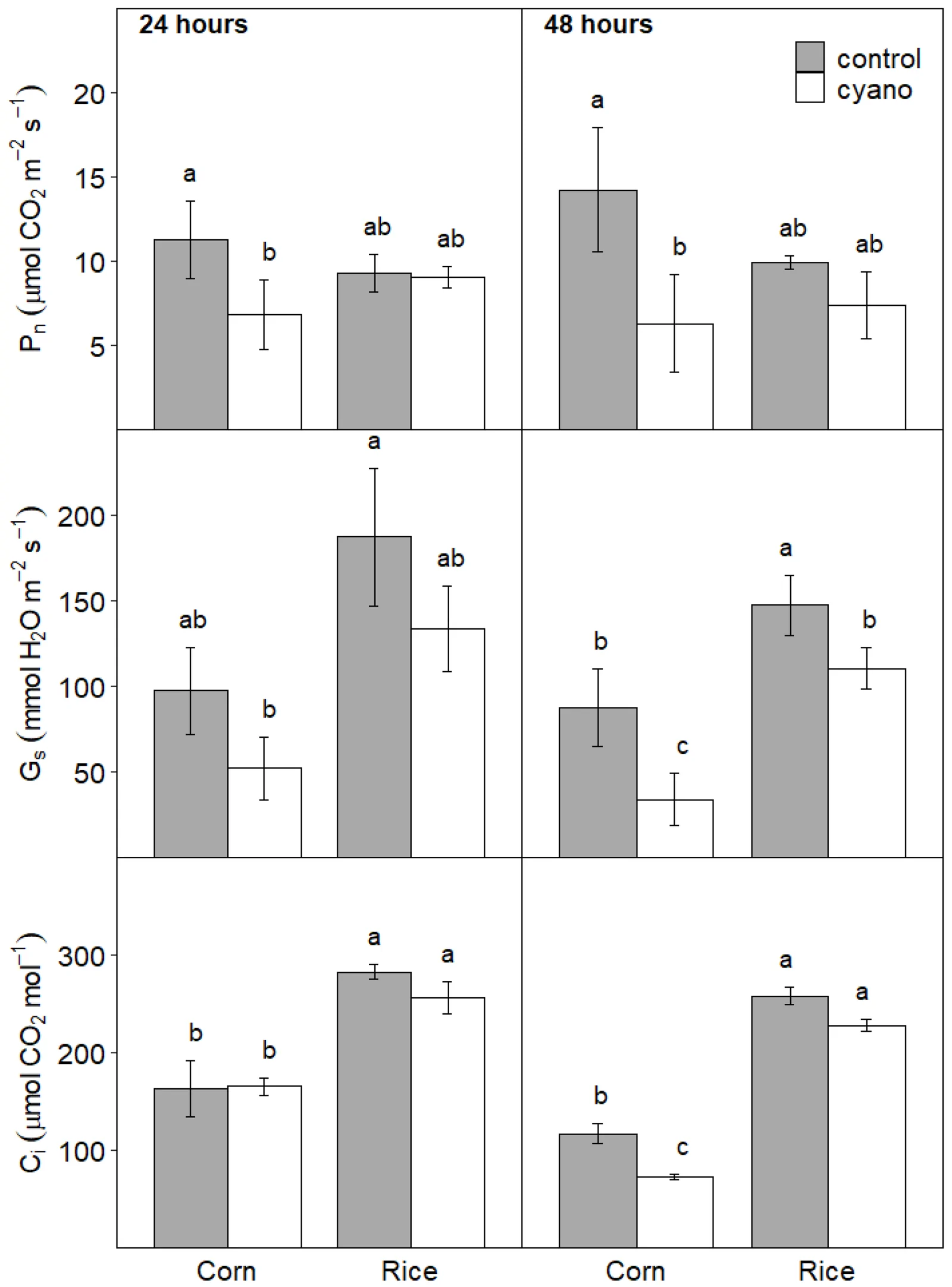
Gas-exchange measurements for corn and rice plants grown in hydroponic nutrient solution (control) or lysed *Anabaena flos-aquae* cells in nutrient solution (cyano) after 24 and 48 h. Bars represent means ± 1 SE for net photosynthesis (P_n_), stomatal conductance (G_s_), and leaf internal CO_2_ concentration (C_i_). Different letters represent pairwise differences in means for each plot.

This study was much shorter than Experiment 1, lasting only 48 h in duration. Corn plants grown hydroponically in cyanobacterial extracts had lower P_n_ compared to control plants after 24 and 48 h (a 39% and 55% decrease, respectively); in contrast, P_n_ in rice plants was not affected at 24 h and decreased only 25% after 48 h (Figure 4). Corn and rice plants had significantly lower G_s_ when grown in extracts for 48 h (61% and 25%, respectively); decreases at 24 h were non-significant (Figure 4). Internal CO_2_ concentrations were similar between control and extract plants of each species after 24 h (Figure 4), indicating that the initial effects of extract on CO_2_ fixation were not due to G_s_. For corn plants, C_i_ was reduced by 54% after 48 h, indicating stomatal closure contributed to reduced P_n_ after 2 d (Figure 4).

**Figure 5:**
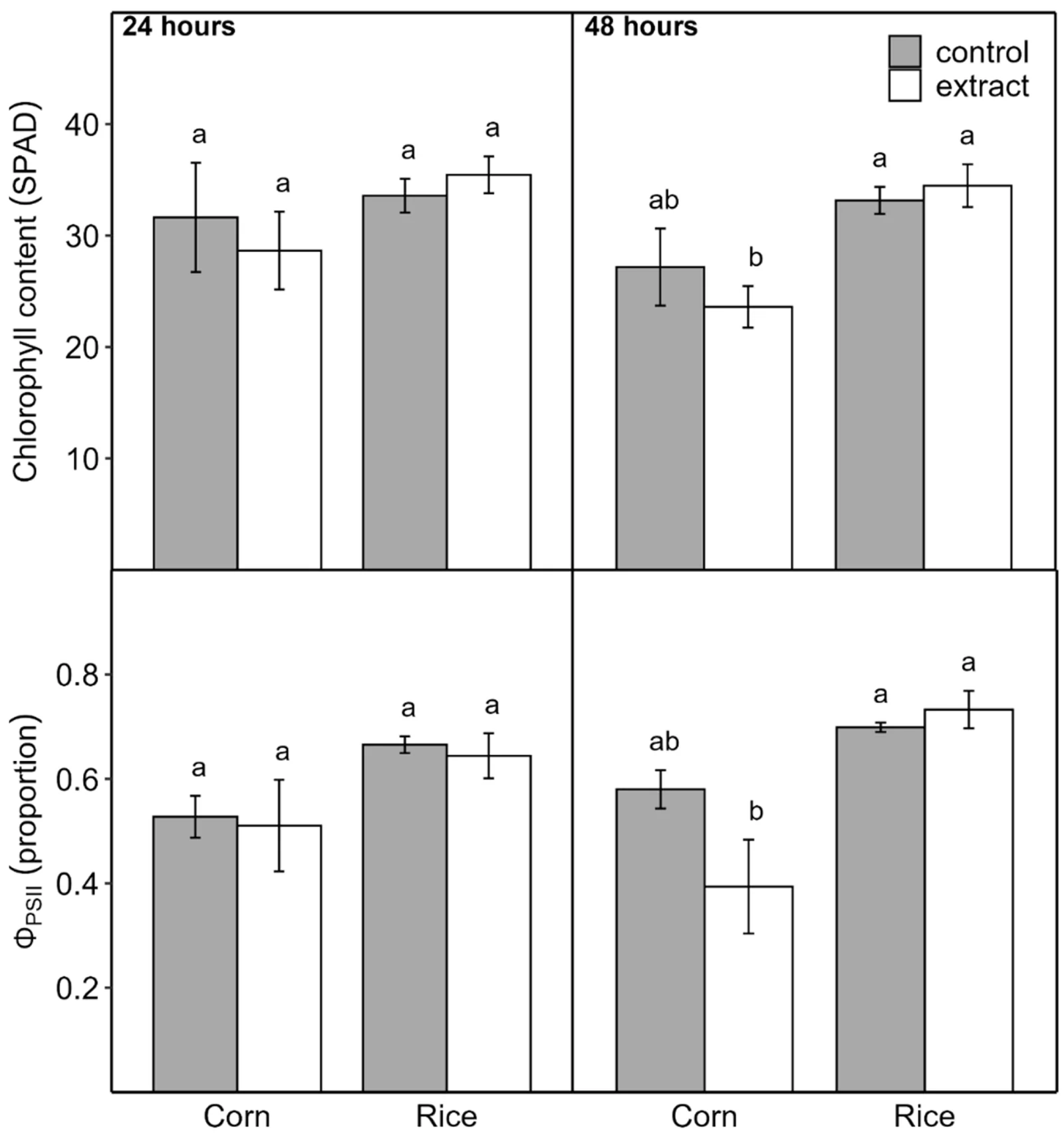
Chlorophyll concentration and photosystem II yield (Φ_PSII_) for corn and rice plants grown in hydroponic nutrient solution (control) or lysed *Anabaena flos-aquae* cells in nutrient solution (extract) after 24 and 48 h. Bars represent means ± 1 SE. Different letters represent pairwise differences in means for each plot.

Chlorophyll content was not significantly affected by extracts in either species in this experiment, although there was a non-significant trend for lower chlorophyll in extract-treated corn plants compared to controls after 48 h (Figure 5). Φ_PSII_ was not affected by extract in rice plants, but it was reduced by 32% in extract-treated corn plants compared to controls after 48 h (Figure 5).

**Figure 6:**
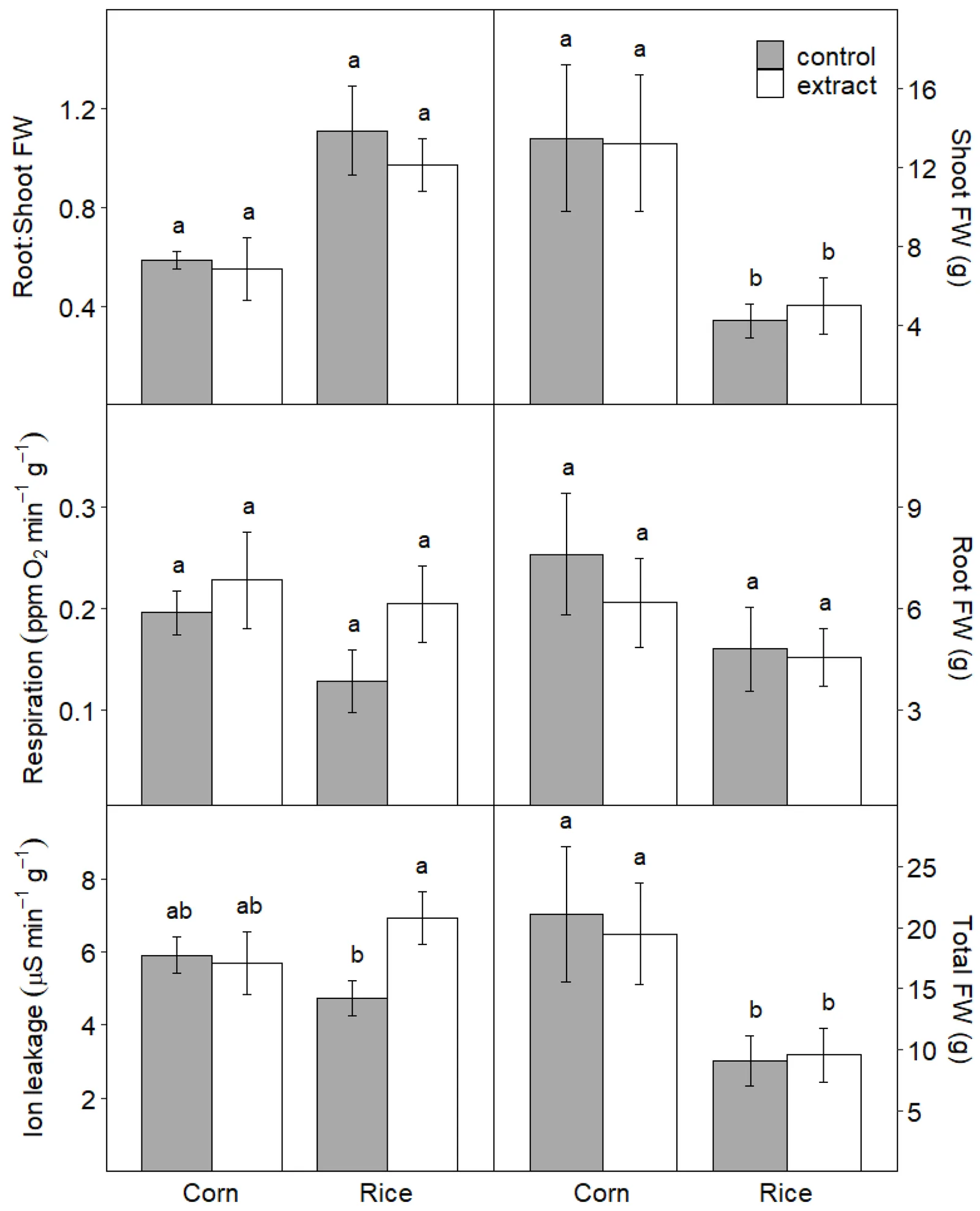
Biomass (fresh weight, FW) and root functional measurements for corn and rice plants grown in hydroponic nutrient solution (control) or lysed *Anabaena flos-aquae* cells in nutrient solution (extract) for three days. Bars represent means ± 1 SE. Different letters represent pairwise differences in means for each plot.

Shoot, root, and total FW were not significantly affected by treatment with cyanobacterial extract for either species after 48 h (Figure 6). The root:shoot mass ratio was also not significantly affected by extract in either species. Differences in root respiration rate were not significant among treatments, due to high variability within treatments, but a non-significant trend towards an increase in root respiration was observed in both species after 48 h (16% and 59% in corn and rice, respectively) (Figure 6). Root ion leakage was similar in extract-treated and control corn plants, but extract-treated rice plants had 46% higher ion leakage rates compared to controls (Figure 6).

### 2.3. Experiment 3: Anatoxin-a in Soil

**Figure 7:**
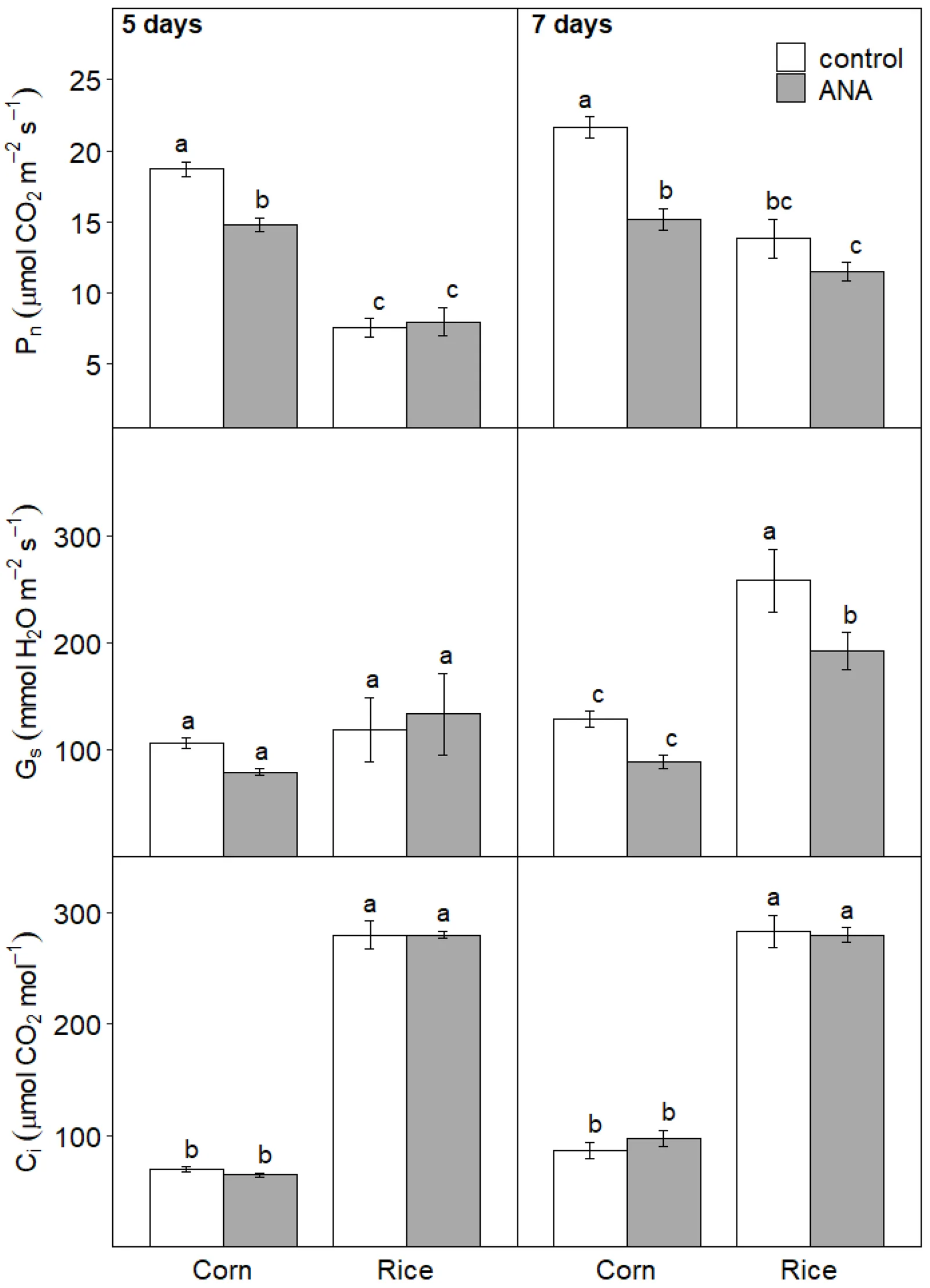
Gas-exchange measurements for corn and rice plants grown in soil and watered with pure water (control) or 2 µM anatoxin-a (ANA) after 5 and 7 days. Bars represent means ± 1 SE for net photosynthesis (P_n_), stomatal conductance (G_s_), and leaf internal CO_2_ concentration (C_i_). Different letters represent pairwise differences in means for each plot.

Corn plants treated with pure anatoxin-a (ANA) had lower P_n_ compared to control plants after both 5 and 7 d of treatment (30% lower at 7 d) (Figure 7). In contrast, P_n_ in rice plants was not affected by ANA after 5 d, and P_n_ decreased (non-significantly) by only 15% after 7 d. Similar trends for G_s_ were observed, with non-significant decreases in ANA-treated corn after both 5 and 7 d, but decreases in ANA-treated rice only after 7 d (Figure 7). Internal CO_2_ concentration was not affected by ANA treatment in either species (Figure 7), indicating, as in Experiments 1 and 2, that the decreases in P_n_ were not caused by stomatal closure.

**Figure 8:**
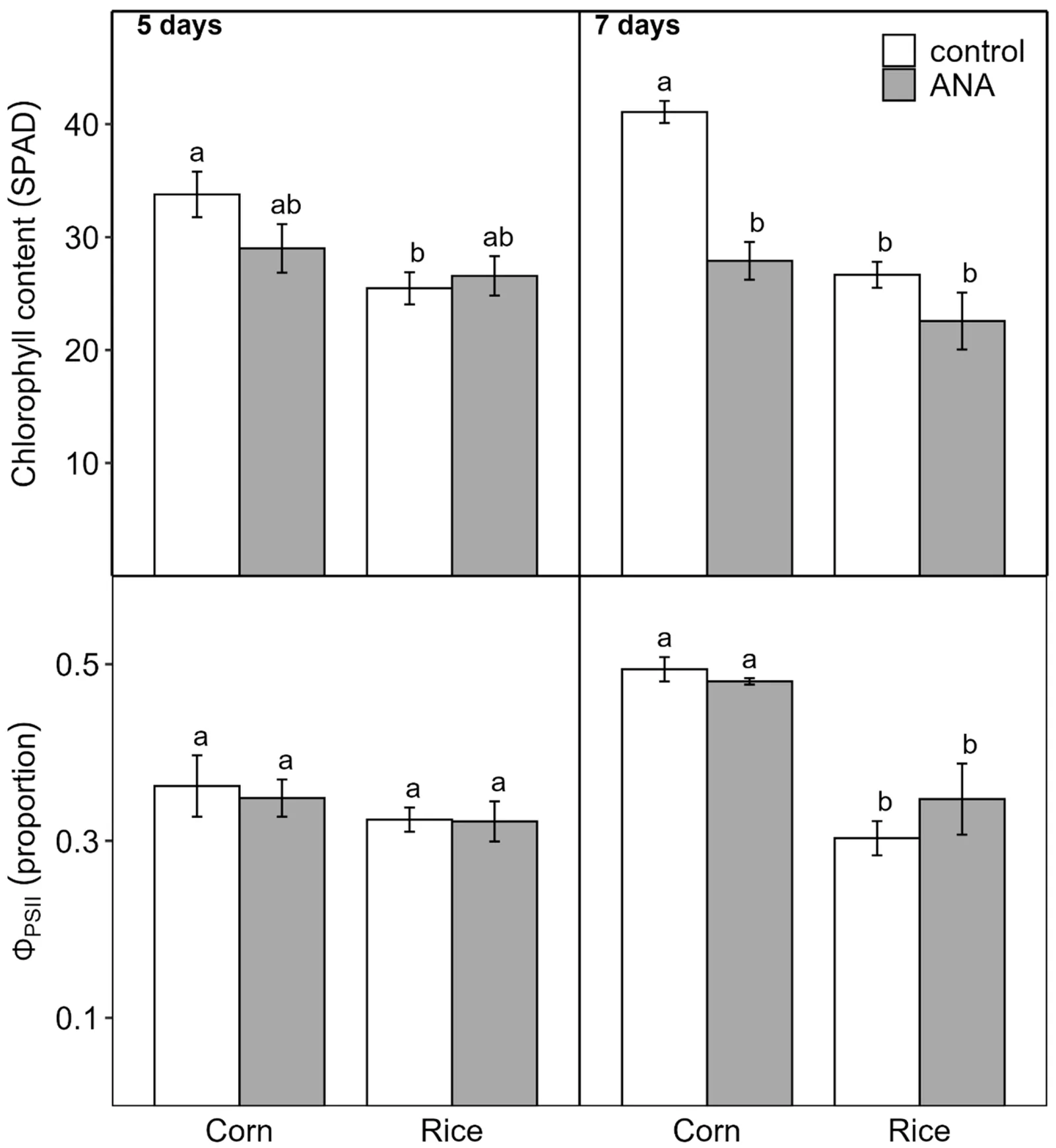
Chlorophyll concentration and photosystem II yield (Φ_PSII_) for corn and rice plants grown in soil and watered with pure water (control) or 2 µM anatoxin-a (ANA) after 5 and 7 days. Bars represent means ± 1 SE. Different letters represent pairwise differences in means for each plot.

Differences in chlorophyll content began to appear in corn plants treated with ANA compared to controls after 5 d, and it was 32% lower in ANA-treated corn after 7 d (Figure 8). In contrast, rice plants had similar chlorophyll content between treatments after 5 and 7 d, with only a 15% lower SPAD value in ANA-treated plants after 7 d. Φ_PSII_ was unaffected by ANA treatment in both species (Figure 8).

**Figure 9:**
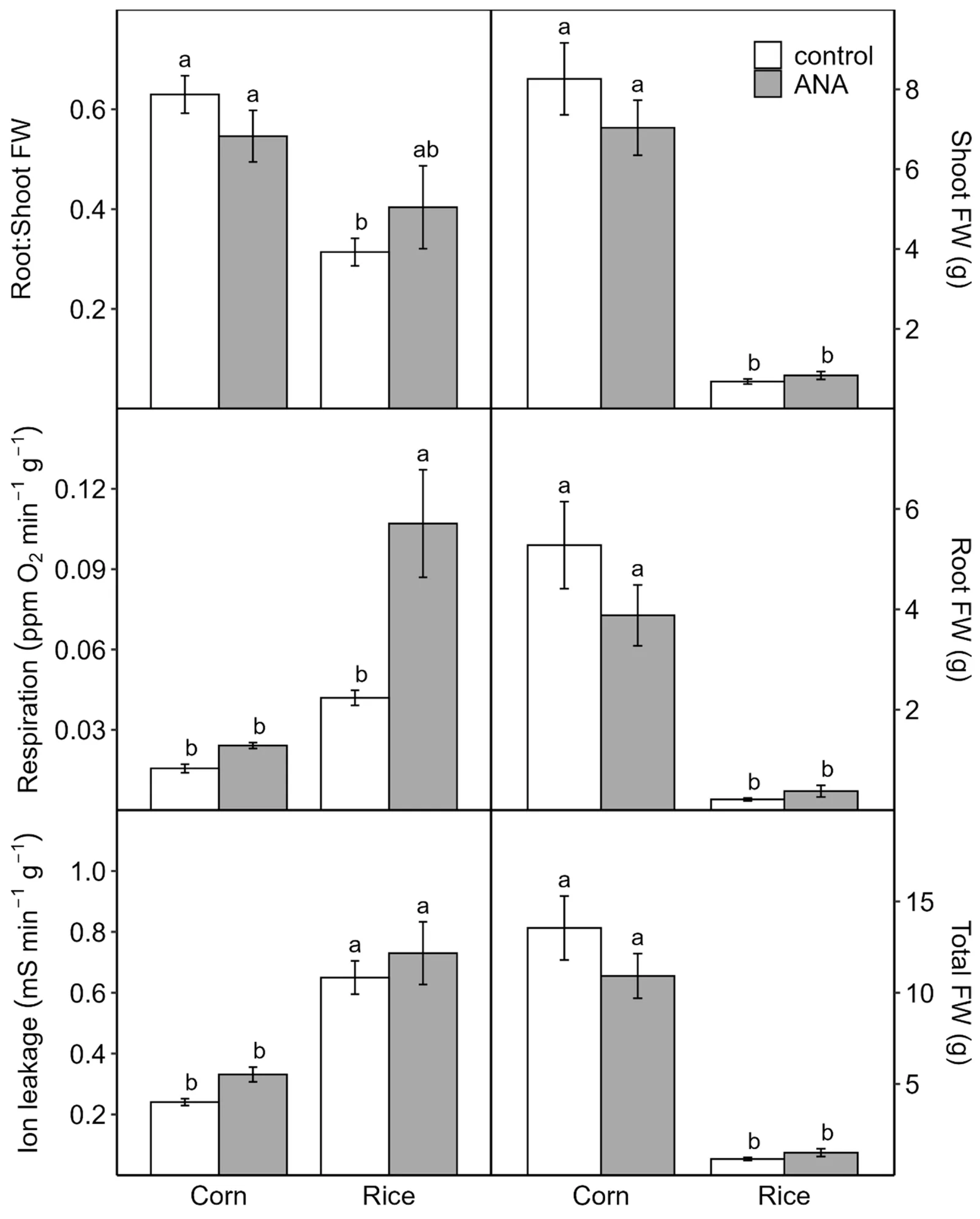
Biomass (fresh weight, FW) and root functional measurements for corn and rice plants grown in soil and watered with pure water (control) or 2 µM anatoxin-a (ANA) for 7 days. Bars represent means ± 1 SE. Different letters represent pairwise differences in means for each plot.

Shoot, root, and total fresh mass of corn and rice were not significantly affected by treatment with ANA after 7 d; however, there was a trend for lower shoot, root, and total biomass in ANA-treated corn compared to untreated controls (14%, 26%, and 19%, respectively) (Figure 9). The root:shoot mass ratio was also not affected significantly by treatment with ANA in either corn or rice. Root respiration rates were similar between treatments for corn, but root respiration was 150% higher in rice plants watered with ANA compared to controls (Figure 9). Root ion leakage was not significantly affected by ANA in either corn or rice (Figure 9).

### 2.4. Across-Experiment Analysis of Cyanotoxin Effects

**Figure 10:**
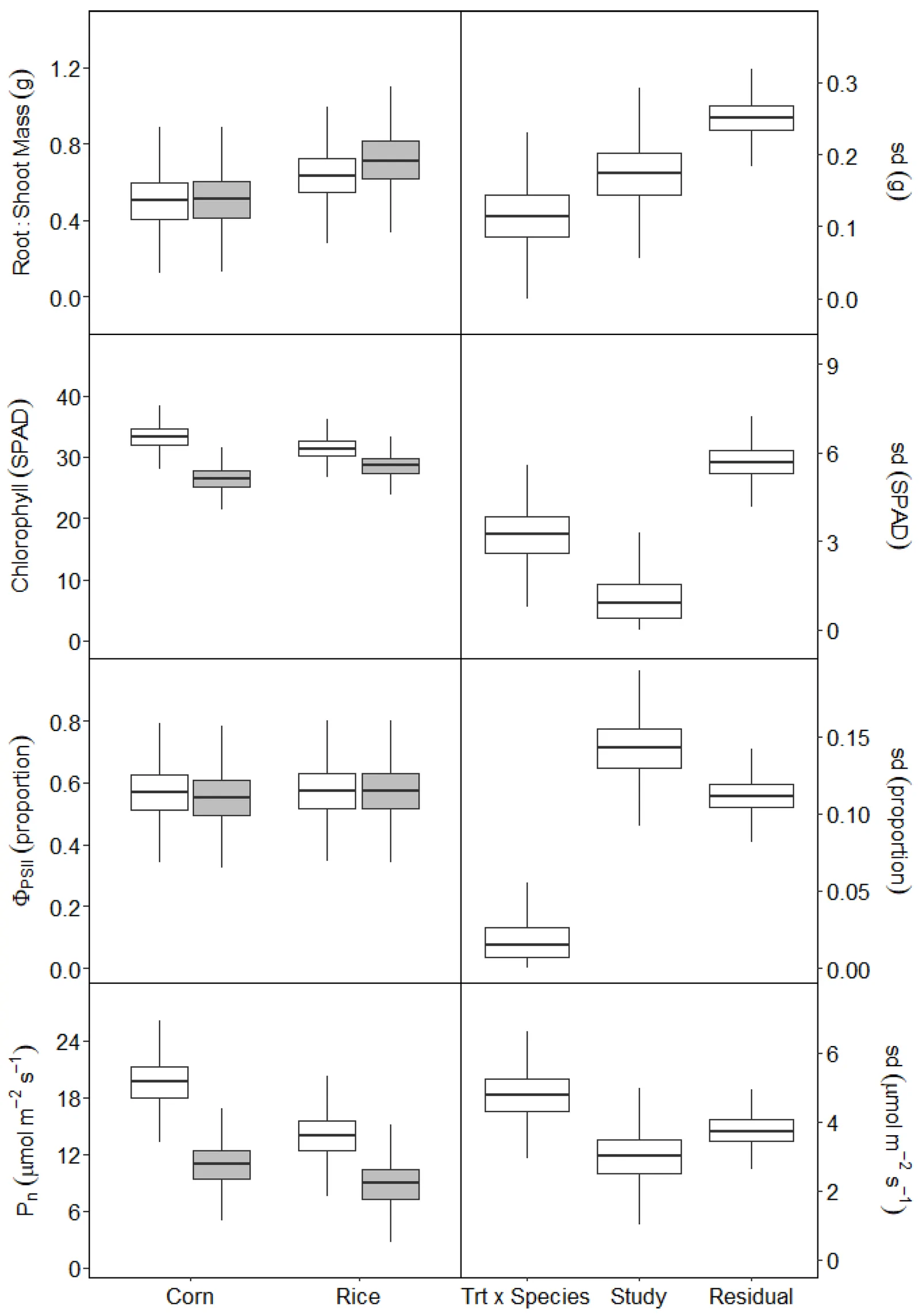
Estimates of effects (**left**) and model variance components (**right**) for root:shoot mass ratio, chlorophyll concentration, PSII yield (Φ_PSII_), and net photosynthesis (P_n_) based on a Bayesian hierarchical linear model built with results from all three experiments. Box plots (with median, 5%, 25%, 75%, and 95% quantiles) represent the distribution of Markov Chain Monte Carlo-simulated responses for each treatment and species combination (**left**), and the model variance distribution (plotted as standard deviation, sd) of each factor included in the model (**right**). Higher variance indicates a stronger influence on the estimated effect distributions. Estimates of effects are split into untreated plant responses (white boxes) and responses of plants treated with cyanotoxins (gray boxes).

When the data from each experiment were combined into a single statistical analysis (using data at final harvest only), some consistent patterns emerged regarding the effects of cyanotoxins (extracts or ANA) on corn and rice function (Figure 10). Across the three experiments, cyanotoxin-treated corn plants were modeled to have a 44% lower rate of Pn compared to controls, whereas rice plants were modeled to have a 36% lower rate of Pn when treated with cyanotoxins compared to controls. In contrast, as also observed in each experiment individually, the model indicated that extracts and ANA had little effect on Φ_PSII_ in either species. Chlorophyll content was predicted to be 20% lower in corn when treated with cyanotoxins, but only 8% lower in toxin-treated rice. The root:shoot mass ratio was modeled to be similar in corn plants regardless of toxin treatment, but slightly increased in rice, by 16%, when treated with cyanotoxins.

The distribution of the variance components for each model reflects the impact of treatment × species and the impact of experiment-of-origin (i.e., Experiment 1, 2, or 3 = “study”) on the modeled results. P_n_ was more influenced by the interactive effect of treatment × species than by the experiment, as shown by the higher standard deviation attributed to treatment × species (Figure 10). In contrast, Φ_PSII_, chlorophyll content, and root:shoot mass ratio were not well-predicted by treatment × species effects, and study had a high degree of variance for Φ_PSII_; hence, their models had high residual variance, indicating the models poorly predict outcomes from the given factors.

## 3. Discussion

### 3.1. Corn Was More Sensitive to Cyanotoxins than Rice

Rice, which is flood tolerant, has a well-developed constitutive exodermis in its roots, which limits entry of water into roots in flooded soils. Corn, which is flood sensitive, lacks the ability to produce a well-developed exodermis. Because of this difference in root anatomy between rice and corn, we hypothesized that rice would be less sensitive to root exposure to cyanobacterial toxins than corn. As hypothesized, across the three experiments, declines in net photosynthesis (P_n_), leaf chlorophyll concentration, and/or biomass occurred sooner, or were larger, for corn than for rice when treated with cell extract from *Anabaena flos-aquae* or from anatoxin-a (ANA). The effects on PSII yield (Φ_PSII_), root function (respiration and ion leakage), and root:shoot mass ratio were smaller or variable across rice and corn. Importantly, negative effects of cyanotoxins on rice were only delayed compared to corn (e.g., one to a few days, depending on the experiment). Our results are consistent with a recent meta-analysis (based on diverse responses categorized as morphology, photosynthesis, or antioxidant metabolism) that found that rice, the only flood-tolerant species in their analysis, was moderately resistant to microcystin (MC) toxins (either *Microcystis* extracts containing MC or pure MC) compared to other crops, and it was slightly more resistant than corn [8]. Also, the wetland plant, *Typha angustifolia*, which grows with its roots submerged in flooded soils and has a well-developed constitutive exodermis, was sensitive to low levels of MC applied to roots [28]. Hence, possession of a well-developed root exodermis did not prevent uptake of either large (MC = ca. 1000 g mol^−1^) or small-sized (ANA = 165 g mol^−1^) cyanotoxins into roots.

Regarding photosynthetic physiology, we found that P_n_, G_s_, and leaf chlorophyll concentration were more sensitive to cyanotoxins than Φ_PSII_. Further, we found that the decreases in P_n_ were not caused by decreases in G_s_, since toxin treatments did not decrease leaf C_i_. Given that Φ_PSII_ was less affected by toxin treatments in our study, it is likely that toxins affected photosynthesis downstream of PSII (i.e., carbon fixation). These results are consistent with our recent work, wherein corn and lettuce (*Lactuca sativa*) exposed to live cyanobacteria, cyanobacterial extracts, or single pure toxins, exhibited decreases in various aspects of photosynthesis (Φ_PSII_, G_s_, chlorophyll, and/or carbon fixation). Specific effects on photosynthesis depended on the mode of exposure (root vs. leaves), growing system (root exposure in soil vs. hydroponics), and toxin provided (extract, MC-LR, ANA, b-methyl-amino alanine, or lipopolysaccharide) [10]. In this previous study, P_n_ and chlorophyll concentration were also more sensitive to toxin exposure than Φ_PSII_, and decreases in P_n_ were mostly attributable to decreases in carbon fixation and chlorophyll rather than stomatal closure.

Across our three experiments, toxins were modeled to decrease P_n_ by 44% in corn and 36% in rice, with larger decreases occurring for extract vs. ANA, and toxins were modeled to decrease chlorophyll concentration by 20% in corn and 8% in rice, with similar decreases in extract and ANA. For comparison, in their meta-analysis, Zhang et al. (2021) found that exposure to MC decreased “photosynthetic efficiency” by ca. 47% in corn and 29% in rice [8]. In their study, “photosynthetic efficiency” included any response variable associated with photosynthetic metabolism, but the data set mostly comprised results for Φ_PSII_ and chlorophyll concentration.

As with P_n_, larger negative effects of toxins on biomass were also observed in corn than in rice in the two longer-duration soil experiments (12 and 7 d for Experiments 1 and 3, respectively; no biomass effects were observed in the 2 d hydroponics experiment). In the two soil-based experiments, both shoot and root biomass were smaller in the toxin treatments in corn, especially the extract treatment (>60% smaller), whereas in rice, ANA did not restrict biomass and extract restricted only shoot mass (38% smaller). In our previous work, corn grown in soil and watered with cyanobacterial extracts also had lower shoot and root masses compared to controls, but the lower shoot mass was proportionate to root mass such that root:shoot mass ratio increased [10]. However, in the current study, the effects of toxins on root:shoot mass ratio were inconsistent and not statistically significant. Similar differences in biomass between corn and rice were observed in Zhang et al. (2021) for MC sensitivity, where “morphology” metrics decreased by 61% in corn but only by 38% in rice (morphology in their study included any response variable that affected growth, including mass, leaf number, shoot or root length, and germination rate) [8].

### 3.2. Cyanobacterial Extracts Affected Roots Differently than Anatoxin-a Alone

By comparing the two soil experiments (Experiment 1, extract vs. Experiment 3, ANA), some important differences in the effects of extract and ANA emerged. First, the suppression of P_n_ in both corn and rice was greater for extract than for ANA alone (Figure 1 and Figure 7). Second, while extract and ANA increased root respiration in both corn and rice, in soil-grown plants, the increase was especially large for extract-treated corn (114% increase) and for ANA-treated rice (160% increase) (Figure 3 and Figure 9). Third, both shoot and root (and hence total) biomass were more negatively affected by treatment with extracts than by ANA, especially in corn (similar but non-significant trends were observed in rice) (Figure 3 and Figure 9). These results indicate that corn and rice often responded differently to ANA vs. extract, and that the extract was more detrimental to photosynthesis and growth. Multiple studies have emphasized the variety of metabolites produced by cHAB-forming species and the likelihood for metabolites and cyanotoxins to have synergistic impacts on plant function [16,17,29,30]. Therefore, it was not surprising that the extract applied in our experiments was more harmful to growth and photosynthesis than supplying a single, pure toxin. Similar results have been reported by others, such as a comparison of *Anabaena* and *Microcystis* extracts vs. four different toxins [10], a review of *Microcystis* extract vs. MC [8], and a comparison of *Microcystis* extract vs. MC at proportionate MC concentrations [31].

### 3.3. Soil vs. Hydroponic Effects

Root systems in hydroponic conditions are architecturally and functionally different than soil-grown roots. Therefore, we expected the effects of cyanotoxins on plant function to differ between soil-grown and hydroponically grown corn and rice. Indeed, for example, G_s_ was more affected in rice, and Φ_PSII_ was more sensitive in corn in the hydroponic-extract experiment compared to the soil-extract experiment (comparing results at 48 h in both experiments). In contrast, however, we observed similar impacts to P_n_ and chlorophyll concentration in both species after 48 h of extract treatment in soil and hydroponics. Given that our soil-extract and hydroponic experiments differed in duration, we cannot compare biomass or root respiration results from the two experiments, as these data were collected at final harvest (12 d vs. 3 d of treatment, respectively). However, in a meta-analysis, Zhang et al. (2021) found that exposure to MC decreased “morphology” and “photosynthetic efficiency” metrics more in hydroponic systems than in soil or solid substrates, although high variability existed in certain culture systems (e.g., in vermiculite or culture medium substrate) [8].

## 4. Conclusions

In this study we compared the physiological responses of two grass species with differing root anatomy, corn and rice, to cyanotoxin exposure, either as lysed-cell extracts or as pure anatoxin-a. This is the first study to compare the response to cyanotoxins of these two species directly. We hypothesized that the root structural differences between corn and rice would have implications for cyanotoxin uptake and therefore the severity of toxic effects, ultimately making corn more sensitive to cyanotoxins than rice due to the presence of a more well-developed constitutive exodermis in rice. Our results identified key differences in the effects of cyanotoxins on corn vs. rice. Photosynthesis, root respiration, and biomass for both corn and rice were impacted by treatment with extracts or anatoxin-a. Although the toxic effects for corn were sometimes more rapidly apparent (e.g., P_n_ and chlorophyll) or severe (e.g., P_n_ and biomass), both species exhibited similar inhibitory effects to extracts and anatoxin-a after three or more days of exposure. Based on these experiments, differences in the tolerances of these two species to cyanotoxins were not the result of toxin exclusion at the root level. Because root morphological traits did not prevent damage from cyanotoxin exposure, we expect that plants with an exodermis will not be protected from cyanotoxins, and exposure is likely to damage photosynthesis and inhibit growth. Further testing is required to understand to what extent the development of a root exodermis impacts toxin uptake. Additionally, plants lacking the ability to make any exodermis, e.g., barley [32], may be less well protected from cyanotoxin exposure than either corn or rice. How cyanotoxins differently affected corn and rice must be due to other physiological or agronomical differences, especially in a field setting, which warrant examination in future work.

## 5. Materials and Methods

### 5.1. Cyanobacterial Growth and Extract Preparation

*Anabaena flos-aquae* (Bornet & Flauhault; synonym: *Dolichospermum flos-aquae*) was purchased commercially (Carolina Biological, Burlington, NC, USA) and grown in 2 L glass flasks containing complete nutrient solution [25% Schenk and Hildebrandt (SH) basal salts (Millipore Sigma, St. Louis, MO, USA) [33] and 4-(2-hydroxyethyl)-1-piperazineethanesulfonic acid (HEPES) (0.5 mM, pH 7) (Millipore Sigma, St. Louis, MO, USA) in deionized (DI) water]. Cultures were grown with constant aeration in a Biosafety-Level-2 hood under constant fluorescent light [ca. 50 µmol m^−2^ s^−1^ photosynthetically active radiation (PAR)] at room temperature (ca. 25 °C). Cultures were allowed to grow for ca. seven days, or until absorbance at 665 nm on a spectrophotometer (UV-1650 PC, Shimadzu, Columbia, MD, USA) reached ca. 0.1 absorbance units. Then, 60% of the culture volume was vacuumed through Whatman GF/F glass microfiber filters (Millipore Sigma, St. Louis, MO, USA) to collect cells. Cells captured on the filters were frozen at −80 °C. The remaining 40% of initial culture volume was refilled with 25% SH salts to its original volume. Cell filtration was repeated weekly.

Frozen cells on filters were re-suspended with 0.1% Tween-20 (Millipore Sigma, St. Louis, MO, USA) in DI water (ca. 1 L), lysed by blending in a kitchen blender with a glass pitcher, and filtered through a cotton cloth to remove cell walls and filter fibers. Filtered solutions were stored at 4 °C until use. Cell extract concentrates were diluted approximately 1:10 in plant nutrient solution (see below), until absorbance at 665 nm on a spectrophotometer read 0.1 units. Dilutions were used for soil and hydroponic experiments.

### 5.2. Plant Growth

*Zea mays* (L.) ‘Solstice’ (Johnny’s Selected Seeds, Winslow, ME, USA) was germinated in the greenhouse in drainable plastic trays filled with calcined clay. *Oryza sativa* (L.) ‘Nipponbare’ (USDA-ARS, Stuttgart, AR, USA) was germinated in plastic trays filled with saturated peat (Lambert, Rivière-Ouelle, QC, Canada) in the greenhouse. Soil pH was between 5.4 and 6, and was near neutral when saturated, which is suitable for corn and rice [34]. Temperatures in the greenhouse typically ranged between 22 and 32 °C. A 15 h photoperiod was maintained during winter using supplemental LED lamps (LED150ED28/740, General Electric, Boston, MA, USA) that added 125 µmol m^−2^ s^−1^ PAR to ambient sunlight (up to 1600 µmol m^−2^ s^−1^ PAR at midday with full sun). Complete nutrients were provided weekly [4.5 mM KNO_3_, 0.5 mM NH_4_NO_3_, 1 mM CO(NH_2_)_2_, 2 mM KH_2_PO_4_, 2 mM CaCl_2_, 1 mM MgSO_4_, 1 mM K_2_SO_4_, 50 µM Fe-EDTA, 50 µM H_3_BO_3_, 10 µM MnCl_2_, 2 µM CuSO_4_, 2 µM ZnSO_4_, and 0.1 µM NaMoO_4_ (pH 6.0 ± 0.1)]. Young plants were watered as needed until they reached the V3 stage (for corn) or the first tillering stage (for rice) before being transplanted into experimental conditions.

### 5.3. Experimental Design

For experiments conducted in pots, corn and rice plants were transplanted into 750 mL square plastic pots (one plant per pot) filled with peat (Lambert) in single-pot plastic catch trays to capture flow-through. In the first pot experiment, corn and rice (five plant replicates per species per treatment) were watered in the greenhouse with 150 mL of cyanobacterial cell extracts or DI water (control) every other day for 12 d. For the second pot experiment, corn and rice pots were moved to a laboratory and placed in a chemical fume hood for treatment with anatoxin-a (ANA). Treatment was conducted in the fume hood to minimize the risk of aerosolized toxin exposure. Lab temperatures ranged from 22 to 26 °C. Relative humidity was 20–25% in the fume hood. Light was provided by a LED growth lamp (Phantom Pheno 4400; Hydrofarm, Shoemakersville, PA, USA), for a 15 h photoperiod, at a light intensity of 900–1100 µmol m^−2^ s^−1^ PAR. Plants were randomly re-positioned within the fume hood daily to account for the spatial light gradient from the lamp. Six replicate plants per species were watered with either 150 mL of 2 µM purified ANA (anatoxin-a fumarate, Cayman Chemical, Ann Arbor, MI, USA) or DI water every other day for 7 d.

For the hydroponic experiment, corn and rice plants (five replicate plants per species per treatment) were transplanted into 500 mL opaque glass jars with roots submerged in 150 mL of either plant nutrient solution (described above, plus 0.01% Tween-20) or cyanobacterial extract and aerated with a bubbler. Plants were transplanted and treated for 48 h in the greenhouse, and volume was maintained at 150 mL by refilling daily with DI water.

In all experiments, plants were measured at multiple time points to monitor gas exchange, chlorophyll content, and chlorophyll fluorescence. Net photosynthesis (P_n_), stomatal conductance (G_s_), and internal CO_2_ concentration (C_i_) of recently expanded leaves were measured with a LICOR 6400XT (LI-COR, Inc., Lincoln, NE, USA; with 2 × 3-cm cuvette and LED lighting) at 400 ppm CO_2_, 28 °C, and 1000 µmol m^−2^ s^−1^ PAR. Leaves that did not fill the cuvette chamber were measured for the actual leaf surface area using a ruler. Relative chlorophyll content (area basis) was measured with a SPAD meter (Spectrum Technologies, Aurora, IL, USA). Chlorophyll fluorescence was measured at growth light intensity using a portable fluorometer (OS1-FL; Opti-Sciences, Hudson, NH, USA) to determine light-adapted photosystem II yield (Φ_PSII_). At the end of each experiment, shoots were separated from roots and measured for fresh weight (FW). Roots were rinsed with water and patted dry with paper towels, then weighed. Roots were immediately placed in individual glass containers, submerged in 100 mL of fully aerated DI water, and the containers were sealed closed with screw-cap lids for 30 min to determine root respiration. After 30 min, containers were unsealed and the water was measured for remaining O_2_ concentration using a portable dissolved oxygen meter (Pentair, Apopka, FL, USA). Root respiration rate was calculated as the difference in O_2_ concentration (from beginning to end) divided by root FW divided by min.

### 5.4. Statistical Analyses

All statistical analyses were performed in R v.4.5.1 [35]. For each experiment, Welch’s *t*-tests were performed for measurements made over time to compare treatment effects for each species at each time point. For measurements made once, a two-way analysis of variance (ANOVA) was conducted to assess the impact of cyanobacterial toxin treatment (control vs. extract or ANA) and species (corn vs. rice), followed by Tukey’s post hoc test for pairwise mean comparisons for significant sources of variation (*p* ≤ 0.05). Assumptions of homogeneity of variance and normality of residuals were tested with Levene’s test and the Shapiro–Wilk test, respectively. Where assumptions of homogeneity of variance were not met, Welch’s ANOVA was performed and compared to traditional ANOVA results. No differences in statistical significance were detected between the two tests, so traditional ANOVA results are presented with corresponding pairwise comparisons.

To compare the overall effects of treatment (control, extract, or ANA) on corn and rice, Bayesian hierarchical modeling was used to analyze the results of all three experiments combined. Using the R packages ‘rstanarm’ [36] and ‘rv’ [37], linear mixed-effects models were fit to assess the effects of two factors (the combined effect of species and treatment and the effect of experiment) on selected variables that were significantly impacted by treatment in ANOVA models. Variables that had substantially different response ranges between hydroponic and soil experiments (i.e., FW and root respiration) were excluded from our results because model convergence for those variables was unachievable. Final-day measurements of P_n_, chlorophyl content, and Φ_PSII_, as well as root:shoot ratio, were each modeled using ‘rstanarm’. For each model, Markov Chain Monte Carlo (MCMC)-assembled posterior distributions were extracted with the rv package. Simulated samplings were plotted as box and whisker plots to represent the estimated distribution of effects for each measured factor level of species × treatment (control corn, toxin corn, control rice, and toxin rice). In addition, the estimated variance of each factor and the residual variance were plotted to show the relative importance of each element for model fit. The more variance attributed to one factor, the stronger the effect of that factor on model predictions.

## Data Availability

The original contributions presented in this study are included in the article. Further inquiries can be directed to the corresponding author.

## Abbreviations

The following abbreviations are used in this manuscript:

cHAB	Cyanobacterial harmful algal bloom
ANA	Anatoxin-a
MC	Microcystin
P_n_	Net photosynthetic rate
G_s_	Stomatal conductance
C_i_	Leaf internal CO_2_ concentration
PAR	Photosynthetically active radiation
Φ_PSII_	Photosystem-II yield
MCMC	Markov Chain Monte Carlo
FW	Fresh weight
